# EQISP: efficient quantized image signal processing with multi-scale pyramid fusion for resource constrained embodied perception

**DOI:** 10.3389/fnbot.2026.1866481

**Published:** 2026-06-08

**Authors:** Tongxin Yang, Ling Guo, Jie Li, Qi Qin, Bowen Liu, Qian Zhang

**Affiliations:** 1 School of Naval Architecture, Ocean and Civil Engineering, Shanghai Jiao Tong University, Shanghai, China; 2 The School of Computer Science and Engineering, Chongqing University of Science and Technology, Chongqing, China; 3 Department of Military Logistics, Engineering University of the PLA Joint Logistic Support Force, Chongqing, China; 4 School of Computer Science and Internet of Things, Chongqing Institute of Engineering, Chongqing, China

**Keywords:** AI-ISP, edge vision, embodied intelligent systems, embodied perception, pyramid fusion, quantization

## Abstract

**Introduction:**

Resource-constrained environmental perception requires autonomous robots and embodied intelligent systems to process visual signals efficiently while preserving image fidelity in complex real-world environments. However, converting high dynamic range RAW sensor data into perceptually faithful RGB images remains computationally expensive, thereby limiting the deployment of neural image signal processors on edge platforms with restricted memory, energy, and computational budgets.

**Methods:**

Consequently, this study proposes the enhanced quantized image signal processor (EQISP), comprising the quantized convolutional neural network (QCNN) and the unified pyramid fusion algorithm (UPFA). QCNN employs dynamic fixed-point hybrid quantization, which adjusts parameter ranges according to the linear relationship between threshold standard deviation and fractional length, thereby significantly reducing the computational load. Meanwhile, UPFA utilizes Gaussian pyramids to capture global illumination and Laplacian pyramids to preserve fine details, enabling multi-scale, multi-exposure fusion and iterative reconstruction to mitigate detail loss induced by quantization.

**Results:**

Comprehensive comparative experiments demonstrated that EQISP achieved a PSNR of 22.90 dB, an SSIM of 0.9278, and 164.843 GFLOPs. Compared with the PyNET baseline, EQISP improved the PSNR by 1.71 dB while reducing the computational cost by a factor of 4.24. Furthermore, deployment experiments on an NVIDIA Jetson TX2 development board showed that EQISP achieved a model size of 57 MB, an inference latency of 189 ms, an inference speed of 6.1 FPS, and a peak memory usage of 2.2 GB.

**Discussion:**

These results provide practical evidence that EQISP can serve as an efficient and scalable visual front end for resource-constrained embodied perception systems.

## 1 Introduction

Autonomous robots and embodied intelligent systems increasingly necessitate visual perception pipelines that achieve an optimal trade-off between computational efficiency and image fidelity in resource constrained real world environments (Gupta et al., 2021). In such systems, visual signals acquired by image sensors must be efficiently transformed into high fidelity representations to support downstream perception, interaction, navigation, and control (Gupta et al., 2021). As a critical front end component of machine vision pipelines, image signal processing (ISP) converts raw sensor measurements into perceptually meaningful RGB images, directly influencing the reliability of subsequent decision making modules (Wang et al., 2024). Recent deep learning techniques have substantially improved ISP performance by training convolutional neural networks (CNNs) on large scale datasets, enabling accurate reconstruction of color, illumination, texture, and high dynamic range details from RAW image data (Santos et al., 2025; Li et al., 2024). However, the high parameter counts, memory access costs, and computational complexity of CNN based ISP models lead to increased latency and energy consumption, thereby hindering their deployment on edge platforms with restricted memory, power, and computational budgets (Sze et al., 2017). To mitigate these constraints, quantization has been widely adopted to reduce storage and computational overhead by representing network parameters and activations with lower numerical precision, including post training quantization schemes that can be efficiently integrated into existing ISP pipelines (Wu et al., 2016). Nevertheless, although quantization improves hardware efficiency, it may compromise the numerical precision required for faithful RAW to RGB reconstruction, creating a critical trade off between computational efficiency and image fidelity in resource constrained embodied perception (Liaw et al., 2002; Chen et al., 2021).

Although quantization algorithms effectively constrain parameter ranges, they may inadvertently alter discrete image representations, risking detail loss (Azuma and Sugie, 2008a,b). Thus, these methods must judiciously balance model complexity and performance to minimize quality degradation amid reduced computational demands. ISP pipelines encompass multiple stages with unique characteristics and requirements; applying uniform quantization often yields suboptimal outcomes, necessitating stage-specific strategies for optimized precision-efficiency trade-offs (Wu et al., 2019). Efficient quantization should incorporate layer-wise adaptability and contextual awareness to enhance dynamism. Overzealous image enhancement can induce distortions and information loss, whereas insufficient processing may perpetuate artifacts and introduce noise, both impairing output fidelity. Prior research mitigates these issues via advanced data preprocessing and streamlined architectures, such as shallower networks or efficient designs. Prevailing techniques include pruning, which eliminates low-impact parameters to curb complexity and overhead; low-rank approximation, decomposing tensors into reduced-rank products for computational savings; and structured matrices, enforcing regularity to further alleviate burdens.

Researchers leverage large training datasets and adaptive strategies within deep learning frameworks to dynamically tune quantization parameters, targeting specific layers for optimized performance across ISP pipeline stages (Wu and Wang, 2018; Farooq and Bazaz, 2020). Transfer learning facilitates fine-tuning of pre-trained features from expansive datasets to domain-specific ISP tasks, particularly valuable amid scarce annotated raw data, thereby accelerating convergence and bolstering generalization. While architectural modifications curtail computational complexity, they frequently erode performance by excising critical parameters, rendering single compression techniques inadequate for network-wide efficacy (Bengio, 2012; Tan et al., 2018). Nonetheless, meticulous data preprocessing and training on heterogeneous datasets safeguard intricate image features, alleviating quantization-induced accuracy degradation, and enabling stage-adaptive optimization to fortify network robustness and adaptability across diverse scenarios.

We propose EQISP, an efficient network, to improve output image quality in deep learning-based ISP tasks. EQISP converts raw images to RGB format with low computational cost and high quality. We introduce a dynamic fixed-point quantization method. This method establishes a linear relationship between the threshold's standard deviation and fractional length. It precisely adjusts the numerical range of network parameters. This balances quantization precision and computational efficiency. To preserve image details, we propose UPFA, which fuses Gaussian and Laplacian pyramids. The Gaussian pyramid captures global illumination. The Laplacian pyramid preserves fine details. The fused image is reconstructed from the global Laplacian pyramid. This retains enhanced details and reduces noise. The key contributions are as follows:

1. We propose the EQISP network, which comprises QCNN and UPFA. It improves image quality in raw-to-RGB conversion while minimizing computational complexity. QCNN reduces network complexity via quantization. UPFA preserves image details through pyramid-based reconstruction. Together, they enable real-time processing of raw images with high accuracy and low computational burden.
2. The QCNN employs dynamic fixed-point hybrid quantization to ascertain the fractional bit length for each parameter, leveraging the linear relationship between the threshold standard deviation and the fractional length, thereby precisely adjusting the parameter value ranges and minimizing the network's floating-point computational load.
3. UPFA algorithm decomposes input images into multi-scale pyramids, employing Gaussian and Laplacian pyramids for consistent feature integration. Through the fusion of images captured at varying exposure levels, effectively retains high-quality details from the original image. Subsequently, the algorithm reconstructs the enhanced image by iteratively applying inverse operations across all pyramid levels.

## 2 Related work

### 2.1 Quantization

Quantization mitigates computational complexity by decreasing the numerical precision of model parameters, thereby enhancing the efficiency of deep learning models. This process typically entails converting high-precision floating-point representations (e.g., 32-bit) to lower-precision integer formats (e.g., 8-bit), which markedly diminishes memory and computational requirements (Ren et al., 2025). Such conversion simplifies calculations by reducing the number of bits necessary for arithmetic operations, consequently lowering the overall computational load. The decrease in precision not only lessens memory usage but also accelerates the inference process by minimizing data transfer and computational overheads (Wu et al., 2016). Various strategies have been devised to reduce resource consumption in deep learning models, including quantization, pruning, which eliminates superfluous parameters, and knowledge distillation, which transfers knowledge from a larger model to a smaller one (Gou et al., 2021). Pruning decreases model complexity by removing redundant parameters or connections that contribute minimally to the model's performance, thus ensuring efficient resource utilization without significant accuracy loss (Chen, 2022). Conversely, knowledge distillation utilizes the insights of a larger, more complex model by training a smaller model to replicate its outputs, thereby reducing computational costs while maintaining comparable performance. Quantization offers a straightforward yet effective method for reducing model size and expediting inference, particularly beneficial in resource-constrained environments (Kang and Gwak, 2020). This technique involves transforming high-precision representations, significantly lessening the memory footprint and accelerating computations. Reductions in precision directly decrease memory usage and computational demands, resulting in faster execution and lower power consumption (Liu et al., 2023b). A significant advantage of quantization is that it does not require additional model training, enabling its direct application to existing models with minimal overhead. This feature facilitates the seamless integration of quantization into pre-trained models, allowing for immediate deployment in production systems. Quantization has emerged as a widely adopted technique in deep learning, particularly for optimizing inference on resource-constrained devices (Jacob et al., 2018). The quantization of deep learning models to alleviate computational load and model complexity remains a prominent area of research, with ongoing efforts focused on enhancing efficiency without compromising performance. Quantization-aware training (QAT) incorporates quantization operations during the training process, allowing the model to adapt to and mitigate quantization-induced errors (Ashkboos et al., 2024). The quantization errors are backpropagated through the network, enabling the optimization of model parameters to minimize the adverse effects of quantization on performance, as demonstrated in recent studies (Shen et al., 2021; Li et al., 2019). This approach optimizes model parameters, mitigating the adverse effects of quantization on performance and ensuring that the model maintains its original accuracy levels despite reduced precision. Learning-based step size quantization incorporates trainable parameters for the step size, enabling the model to dynamically adjust quantization levels during training (Oh et al., 2022; Fan et al., 2024). These adjustable parameters refine the quantization process, minimizing errors and preserving model performance across diverse tasks. Traditional quantization techniques, including uniform and non-uniform quantization, are frequently employed in deep learning models to decrease computational load and enhance inference speed (Allwin et al., 2024). Uniform quantization utilizes a constant step size across the entire value range, which simplifies the quantization process but may result in accuracy loss in certain scenarios. Conversely, non-uniform quantization employs adaptive step sizes, optimizing the quantization process by aligning step size adjustments with the distribution of parameter values (Bondarenko et al., 2024). This method responds to the underlying distribution of model parameters, reducing accuracy loss by applying smaller quantization steps where necessary.

### 2.2 Multi-frame exposure fusion algorithm

Multi-frame exposure fusion algorithms are extensively employed to produce high dynamic range (HDR) images by effectively combining multiple exposures taken under varying lighting conditions (Catley-Chandar et al., 2022; Biswas et al., 2020). These algorithms integrate images captured with different exposure settings, thereby preserving both highlight and shadow details for a more complete representation of the scene. This process maintains intricate highlight and shadow details, which are essential for capturing the full dynamic range of a scene (Keerativittayanun et al., 2021). Consequently, it generates images with an expanded dynamic range, facilitating a more accurate depiction of both bright and dark areas within the scene (Yang et al., 2019). Previous studies have introduced a robust HDR fusion technique that utilizes order statistics, which sorts input images according to pixel intensities and identifies key frames for merging. This method ensures that the most relevant details are retained in the final fusion result by selecting images based on their pixel intensities. The approach extracts critical details from the chosen frames, effectively preserving vital features such as textures, edges, and contrast (Hu et al., 2022). Ultimately, this technique produces high-quality HDR images that enhance visual quality and accurately represent dynamic range (Oh et al., 2014). Multi-frame exposure fusion techniques have been widely utilized in various image processing applications, including scene enhancement, tone mapping, and image alignment. An adaptive exposure fusion method utilizes weighted maps to calibrate the contribution of each input image during the fusion process, thereby ensuring an optimal high dynamic range (HDR) image output. This technique enhances HDR image generation by assigning different weights to the input images based on their respective exposure levels and quality. Adaptive weights are dynamically adjusted according to the quality and exposure levels of each frame (Chen et al., 2023). Additionally, the method modifies fusion parameters based on the weight maps, facilitating precise control over the contribution of each image to the final fusion result. This adaptive strategy improves overall fusion quality by optimizing the balance between highlight and shadow details, resulting in a more realistic and visually appealing HDR image (Liu et al., 2023a; Kong et al., 2024). The approach guarantees a balanced representation of both highlight and shadow regions, preserving fine details in these critical areas while avoiding the introduction of noticeable artifacts (Patel et al., 2015).

## 3 Proposed method

**Figure 1:**
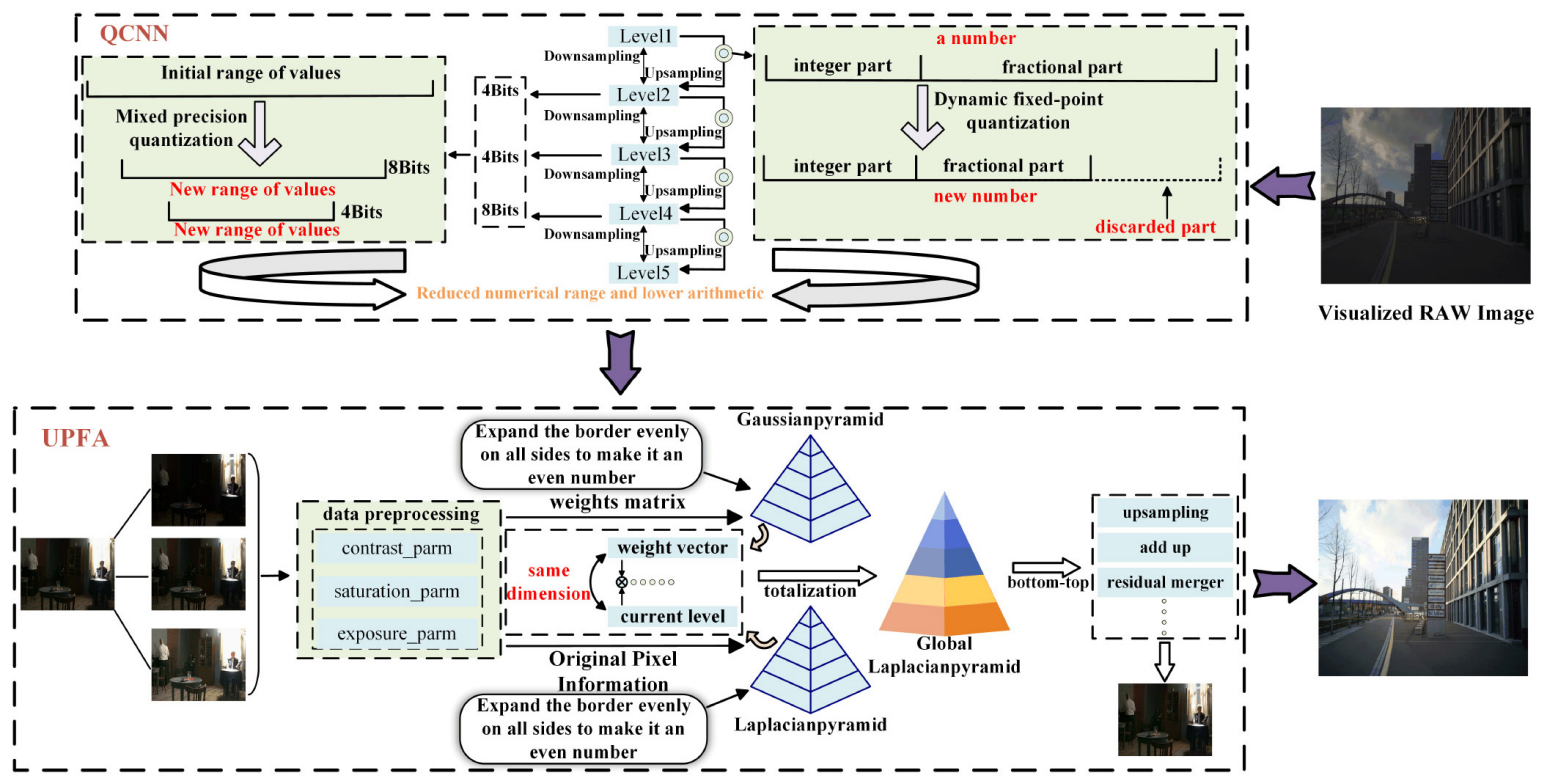
Proposed EQISP network architecture.

The overall workflow of the EQISP is as follows: A single input RAW image is first processed by the QCNN to generate an intermediate RGB output with reduced computational complexity. Subsequently, multi-exposure variants are produced from this RGB output via bracketing simulation and fused using the UPFA to yield the final high-quality RGB image, illustrated in Figure 1. The bracketing simulation algorithm relies on gamma adjustment:


$$\begin{array}{l} I_{k}\left(i,j\right)={\left[I\left(i,j\right)\right]}^{\gamma_{k}} \end{array}$$ (1)

where γ_*k*_∈{0.5, 1.0, 2.0} correspond to high, normal, and low exposure variants, respectively (Equation 1). The algorithmic novelty of EQISP lies in the task-specific integration and adaptation of these components for quantized neural ISP. The fixed-point quantization in QCNN is not implemented as a global uniform quantizer; instead, the fractional length is dynamically determined for each layer according to the statistical dispersion of weights and activations. The pyramid fusion module is also not used as a generic exposure fusion post-processing method. Rather, it is applied to simulated multi-exposure variants generated from the quantized ISP output to compensate for detail attenuation and local contrast degradation caused by quantization. In this way, EQISP forms a unified efficiency-fidelity optimization framework for RAW-to-RGB reconstruction under resource-constrained deployment conditions.

### 3.1 Quantization of convolutional neural networks

**Figure 2:**
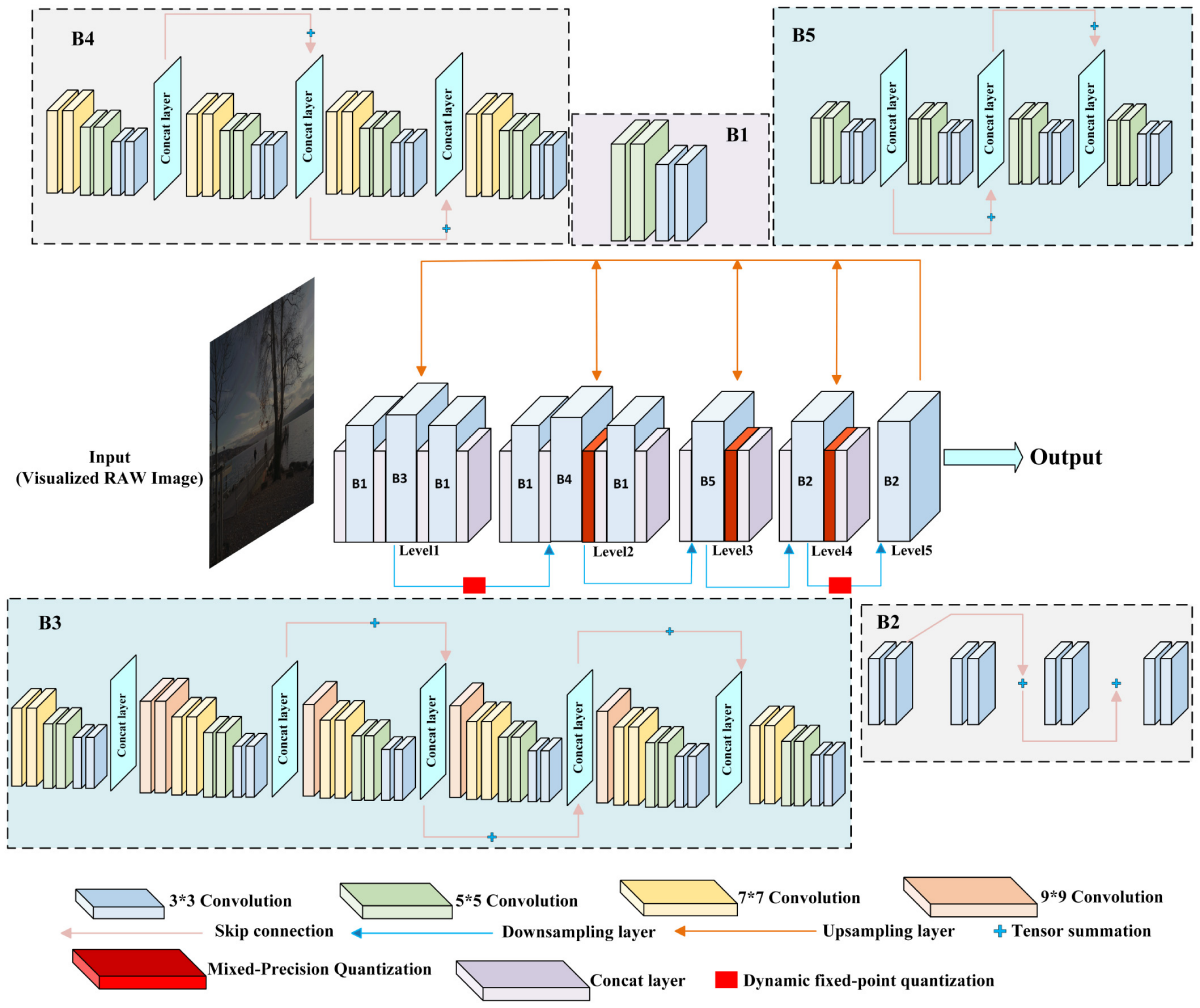
The architecture of the proposed QCNN model.

Figure 2 illustrates the QCNN architecture. It processes images at five scales. QCNN consists of multiple blocks. These apply convolutional filters of varying sizes to feature maps simultaneously. The network merges outputs from these convolutional layers. This captures diverse features at each scale. Levels 4 and 5 focus on global color, brightness adjustments, and gamma adjustments. Training minimizes mean squared error (MSE). Levels 2 and 3 enhance object color and shape characteristics. They also integrate semantic details. Training at these levels combines VGG-based perceptual loss with MSE in a 4:1 ratio. Level 1 operates on the original image. It emphasizes local enhancements, including texture refinement, noise reduction, and color adjustments. Training at this level blends structural similarity index (SSIM), MSE, and VGG-based losses:


$$\begin{array}{l} L=L_{VGG}+0.75*L_{SSIM}+0.05*L_{MSE} \end{array}$$ (2)

The SSIM loss function is designed to enhance the dynamic range of the reconstructed image, while the incorporation of MSE loss serves to mitigate significant color deviations. The weighting coefficients in Equation 2 were determined through preliminary sensitivity experiments rather than being optimized on the test set. Specifically, the coefficient of the VGG perceptual loss was fixed to 1.0 as the main perceptual reconstruction term. We then adjusted the weights of the SSIM and MSE losses to balance structural fidelity, perceptual quality, and pixel-level color consistency. A relatively large SSIM weight was used because structural consistency and local contrast preservation are important for RAW-to-RGB reconstruction. However, assigning an excessively large weight to SSIM may overemphasize local contrast and lead to unstable color reconstruction. Therefore, the SSIM weight was set to 0.75 to strengthen structural preservation while maintaining stable optimization. The MSE loss was assigned a smaller weight of 0.05 because it mainly serves as a pixel-level regularization term to reduce large color deviations. In our preliminary experiments, a larger MSE weight tended to produce over-smoothed results and weakened high-frequency details, whereas a very small MSE weight was less effective in constraining color shifts. In deep learning frameworks, images are represented as multi-dimensional tensors, typically denoted as **x**∈ℝ^*C*×*H*×*W*^, where *C* represents the number of channels, *H* the height, and *W* the width. Each element *x*_*i*_ in this tensor encodes pixel intensities, thereby facilitating spatial hierarchies and channel-wise feature extraction, which in turn enable efficient convolutional operations and backpropagation. To convert the floating-point operations in neural networks to fixed-point representations, the statistical properties of **x** are initially characterized by computing the mean μ and standard deviation σ:


$$\begin{array}{l} \mu =\frac{1}{N}\overset{N}{\underset{i=1}{\sum }}x_{i},\;\sigma =\sqrt{\frac{1}{N}\overset{N}{\underset{i=1}{\sum }}{\left(x_{i}-\mu \right)}^{2}} \end{array}$$ (3)

where *N* = *C*×*H*×*W* represents the total number of elements (Equation 3). These estimators yield unbiased measures of central tendency and dispersion, assuming independent and identically distributed samples—a condition that approximates the behavior of pixel values in natural images owing to local correlations and preprocessing normalization. The data **x** approximately follows a normal distribution $N\left(\mu ,\sigma^{2}\right)$, as supported by the central limit theorem. The initial quantization range is derived from Gaussian principles to encompass the majority of values, thereby establishing a baseline for subsequent refinement. Specifically, the initial minimum and maximum values are set as follows:


$$\begin{array}{l} \underset{\text{init}}{\min}=\mu -3\sigma ,\;\underset{\text{init}}{\max}=\mu +3\sigma \end{array}$$ (4)

This interval (Equation 4) is theoretically grounded in the three-sigma rule, which stems from the properties of the Gaussian probability density function $f\left(z\right)=\frac{1}{\sqrt{2\pi }}e^{-z^{2}/2}$, where *z* = (*x*−μ)/σ. The cumulative probability within the interval [μ−3σ, μ+3σ] is as follows:


$$\begin{array}{l} P\left(|X-\mu |\leq 3\sigma \right)=\int_{-3}^{3}f\left(z\right)dz \end{array}$$ (5)

In a normal distribution (Equation 5), approximately 99.7% of the data values lie within ±3σ of the mean, thereby mitigating the impact of outliers while minimizing information loss. To ensure that the quantization range fully encompasses all observed data points without truncation, the initial bounds are compared with the empirical extrema:


$$\begin{array}{l} \underset{x}{\min}=\min\left\{x_{i}|i=1,\ldots ,N\right\} \end{array}$$ (6)

$$\begin{array}{l} \;\underset{x}{\max}=\max\left\{x_{i}|i=1,\ldots ,N\right\} \end{array}$$ (7)

The (Equations 6 and 7) final quantization bounds are then adjusted as:


$$\begin{array}{ll} \underset{x}{\min} & =\min\left(\underset{\text{init}}{\min},\underset{x}{\min}\right)=\min\left(\mu -3\sigma ,\underset{x}{\min}\right) \end{array}$$ (8)

$$\begin{array}{ll} \underset{x}{\max} & =\max\left(\underset{\text{init}}{\max},\underset{x}{\max}\right)=\max\left(\mu +3\sigma ,\underset{x}{\max}\right) \end{array}$$ (9)

This adjustment ensures that $\underset{x}{\min}\leq \overset{\text{emp}}{\underset{x}{\min}}$ and $\underset{x}{\max}\geq \overset{\text{emp}}{\underset{x}{\max}}$, thereby (Equations 8 and 9) encompassing all data points while incorporating padding when the empirical range is narrower than 6σ or extending the bounds if outliers exceed the initial Gaussian limits. Consequently, μ and σ directly influence $\underset{x}{\min}$ and $\underset{x}{\max}$ by establishing a minimum range width of 6σ centered at μ, thereby adapting to the actual spread of the data. Quantization maps floating-point values to a discrete integer set within a bit width of *b*, thereby reducing storage requirements to *b* bits per element and facilitating integer-based arithmetic for enhanced computational efficiency. The scale factor *S*_*m*_, which normalizes values to the full *b*-bit unsigned integer range [0, 2^*b*^−1], is given by:


$$\begin{array}{l} S_{m}=2^{b}-1 \end{array}$$ (10)

Quantization maps values as Equation 10:


$$\begin{array}{l} \begin{array}{l} Q\left(x\right)=round\left(\frac{x-\underset{x}{\min}}{\underset{x}{\max}-\underset{x}{\min}}\times S_{m}\right)\\ \;/S_{m}\times \left(\underset{x}{\max}-\underset{x}{\min}\right)+\underset{x}{\min} \end{array} \end{array}$$ (11)

Dynamic fixed-point quantization (Equation 11) is employed during forward propagation to reduce memory usage and accelerate inference. This approach maintains a constant word length *WL*, which denotes the total number of bits assigned to each value, while dynamically determining the fractional length *FL*, which controls the number of bits allocated to the fractional part. The value of *FL* determines the trade-off between numerical precision and representable dynamic range. Specifically, a larger *FL* leads to a smaller quantization step and thus higher fractional precision, but it reduces the available integer range and increases the risk of saturation. Conversely, a smaller *FL* enlarges the representable range but results in coarser quantization. To determine *FL*, we match the representable range of the fixed-point format with the statistically dominant range of the weights and activations in each layer. For each tensor, the standard deviation σ is first computed to characterize its dispersion. The clipping boundary is then defined as *p* = 3σ, following the three-sigma principle under the approximate Gaussian assumption discussed above. This boundary covers approximately 99.7% of the values for a normally distributed variable, thereby preserving the dominant numerical range while reducing the influence of rare outliers. A smaller boundary may clip informative values, whereas a larger boundary would allocate more bits to the integer part and reduce fractional precision. Therefore, *p* = 3σ provides a practical trade-off between saturation avoidance and quantization precision. For a fixed-point number, the original value is scaled by 2^*FL*^ before being mapped to the integer domain. Therefore, the quantization step is 2^−*FL*^. For signed tensors, the integer representation range is [−2^*WL*−1^, 2^*WL*−1^−1]. To avoid saturation for values within the selected boundary [−*p, p*], the following condition should be satisfied:


$$\begin{array}{l} p\cdot 2^{FL}\leq 2^{WL-1}-1. \end{array}$$ (12)

Thus (Equation 12), the maximum feasible fractional length is derived as:


$$\begin{array}{l} FL=\left\lfloor \log_{2}\left(\frac{2^{WL-1}-1}{p}\right)\right\rfloor \end{array}$$ (13)

$$\begin{array}{l} FL=\left\lfloor \log_{2}\left(\frac{2^{WL}-1}{p}\right)\right\rfloor \end{array}$$ (14)

The optimal FL (Equations 13 and 14) is determined based on statistical analysis of fixed-point representations. The scaling factor *S*_*n*_ is defined as follows:


$$\begin{array}{l} S_{n}=2^{FL} \end{array}$$ (15)

Values (Equation 15) are shifted left by FL bits to obtain an integer representation. The range bounds are defined as follows:


$$\begin{array}{l} \begin{array}{l} \text{Signed:}\underset{\text{val}}{\max}=2^{WL-1}-1,\;\underset{\text{val}}{\min}=-2^{WL-1}\\ \text{Unsigned:}\underset{\text{val}}{\max}=2^{WL}-1,\;\underset{\text{val}}{\min}=0 \end{array} \end{array}$$ (16)

The input tensor is multiplied by the scaling factor and subsequently clipped (Equation 16), thereby imposing appropriate value constraints:


$$\begin{array}{l} S_{x}=x*S_{2} \end{array}$$ (17)

$$\begin{array}{l} clip_{x}=clamp\left(S_{x},min_{val},max_{val}\right) \end{array}$$ (18)

According to Formula (Equations 17 and 18), Dequantization restores approximate floating-point values (Equation 19):


$$\begin{array}{l} Dq\left(x\right)=\frac{1}{2^{FL}}round\left(clip\left(x*2^{FL},0,2^{WL}-1\right)\right) \end{array}$$ (19)

### 3.2 Uniform pyramid fusion algorithm

The Uniform Pyramid Fusion Algorithm (UPFA) employs multi-resolution pyramid decomposition to integrate information from the input images. Theoretically, this pyramid-based approach leverages the sensitivity of multi-scale features, as modeled in scale-space theory, wherein coarser levels capture global luminance, while finer levels encode local contrast. For each input image *I*_*k*_ (where *k* = 1, …, *N* represents the exposure variant), pixel values *p*—typically encoded in an 8-bit integer range [0, 255] within standard imaging pipelines—are normalized to the unit interval [0, 1] to standardize processing across different exposures and mitigate scale-dependent biases in weight computations:


$$\begin{array}{l} p_{ij}=\frac{p}{255} \end{array}$$ (20)

where *i, j* denote the row and column indices, respectively (Equation 20). This linear normalization preserves relative intensities while converting the data to a floating-point format suitable for subsequent filtering and fusion operations. Theoretically, this step corresponds to affine transformations in signal processing, thereby ensuring invariance under uniform scaling. To facilitate seamless recursive downsampling in the generation of the Gaussian pyramid, where each level halves the resolution, the image borders are extended to ensure even dimensions at every pyramid level, thus preventing artifacts arising from fractional resizing. For an original image with width *w* and height *h*, and with *n* pyramid levels [typically *n* = ⌊log_2_min(*w, h*)⌋+1 to reach a 1 × 1 apex], the adjusted dimensions are determined by ensuring divisibility by 2^*n*−1^:


$$\begin{array}{l} w_{new}=\left\lceil \frac{w}{2^{n-1}}\right\rceil *2^{n-1}h_{new}=\left\lceil \frac{h}{2^{n-1}}\right\rceil *2^{n-1} \end{array}$$ (21)

This ceiling operation determines the smallest multiple of 2^*n*−1^ that is greater than or equal to the original dimensions, followed by border replication to pad the differences *w*_new_−*w* and *h*_new_−*h*. Subsequently, quality weights are computed on a per-pixel basis to direct the fusion process, thereby emphasizing regions exhibiting superior attributes across different exposures. The contrast weights *C*_*k*_ for image *I*_*k*_ quantify local sharpness through the absolute response of a Laplacian filter applied to its grayscale version *I*_*k*, gray_:


$$\begin{array}{l} C\left(i\right)=|imfilter\left(I_{gray},H\right)| \end{array}$$ (22)

where * denotes convolution (Equation 21), and *H* is the discrete Laplacian kernel, typically defined as a second-difference operator (Equation 22). This formulation stems from the Laplacian operator $\nabla^{2}f=\frac{\partial^{2}f}{\partial x^{2}}+\frac{\partial^{2}f}{\partial y^{2}}$, which approximates second-order derivatives and thereby highlights edges and textures as high-frequency components. In the continuous domain, convolution with *H* yields $\nabla^{2}I_{k,\text{gray}}$, with the absolute value ensuring non-negative weights. The saturation weights *S*_*k*_ assess color vividness by computing the root-mean-square deviation of the RGB channels from their local mean $q=\frac{1}{3}\left(I_{k,R}+I_{k,G}+I_{k,B}\right)$:


$$\begin{array}{l} S\left(i\right)=\sqrt{\frac{1}{3}\underset{c\in R,G,B}{\sum }{\left(I_{c}-q\right)}^{2}} \end{array}$$ (23)

The exposure weights *W*_*k*_ evaluate the optimality of pixels around mid-tone values through the product of Gaussian functions across color channels, with a variance of σ^2^ (typically σ = 0.2 based on empirical findings):


$$\begin{array}{l} W\left(i\right)=\underset{c\in R,G,B}{\prod }exp\left(-\frac{{\left(I_{c}-0.5\right)}^{2}}{2\sigma^{2}}\right) \end{array}$$ (24)

This (Equations 23 and 24) multiplicative form ensures joint suitability across channels, arising from independent Gaussian assumptions for each channel; the exponent represents the negative Mahalanobis distance in a univariate normal distribution $N\left(0.5,\sigma^{2}\right)$. The aggregate weight map for each image is typically formulated as the product $W_{k,\text{agg}}=C_{k}^{\alpha }\cdot S_{k}^{\beta }\cdot W_{k}^{\gamma }$, where the exponents α, β, and γ adjust the emphasis and are often set to 1 for balance, although the provided text suggests potential separate or combined applications. These weights are normalized across exposures to yield a convex combination:


$$\begin{array}{l} W_{k,\text{norm}}=\frac{W_{k,\text{agg}}}{\sum_{m=1}^{N}W_{m,\text{agg}}+\epsilon } \end{array}$$ (25)

where ϵ>0 prevents division by zero in uniform regions (Equation 25). Multi-resolution fusion utilizes Gaussian pyramids $G_{k}^{l}$ for each *I*_*k*_ at levels *l* = 0, …, *n*−1, which are constructed through iterative low-pass filtering and downsampling by a factor of 2:


$$\begin{array}{l} G_{k}^{l+1}={↓}_{2}\left(G_{k}^{l}*g\right) \end{array}$$ (26)

where *g* represents a binomial filter approximating a Gaussian (Equation 26), and ↓_2_ denotes decimation. Laplacian pyramids $L_{k}^{l}$ isolate band-pass details:


$$\begin{array}{l} L_{k}^{l}=G_{k}^{l}-{↑}_{2}\left(G_{k}^{l+1}\right)*g \end{array}$$ (27)

with ↑_2_ denoting bilinear upsampling (Equation 27). This decomposition separates frequencies, as demonstrated in Burt and Adelson's framework, wherein $L_{k}^{l}$ captures details at a scale of 2^*l*^. The fused Laplacian $L_{r}^{l}$ is obtained as the weighted sum:


$$\begin{array}{l} L_{r}^{l}=\overset{N}{\underset{k=1}{\sum }}W_{k,\text{norm}}^{l}\cdot L_{k}^{l} \end{array}$$ (28)

with weights potentially computed at each level for refinement (Equation 28). Reconstruction produces the output *R* by summing from the coarsest level:


$$\left\{ \begin{array}{l} R^{n-1}=G_{r}^{n-1}\\ R^{l}=L_{r}^{l}+{↑}_{2}(R^{l+1})*g\text{ for}\;\text{l=n }-\text{ 2},\ldots ,\text{0} \end{array}\right.$$ (29)

where *R* = *R*^0^ denotes the final reconstructed image (Equation 29), and the upsampling operator ↑_2_ is applied to level-consistent fused components to mitigate aliasing.

## 4 Experiments

### 4.1 Training settings and dataset pre-processing

In this section, we comprehensively evaluate the effectiveness of the proposed EQISP framework through a series of experiments. The parameter settings and device information relevant to the EQISP network during the experimental process are as follows:

- Framework and hardware: The EQISP network is implemented in the PyTorch framework (version 1.10.0, Python 3.8 on Ubuntu 20.04, with CUDA 11.3). All training procedures are executed on an NVIDIA Tesla V100 GPU to ensure a consistent and reproducible computational environment.
- Optimizer: We employ the Adam optimizer with a decoupled weight decay of 10^−4^ to promote numerical stability and prevent overfitting.
- Learning rate schedule: The initial learning rate is set to 10^−5^. To ensure stable convergence, the learning rate is progressively adjusted following a cosine annealing learning rate schedule.
- Batch size: To balance computational efficiency and gradient estimation accuracy, the batch size is set to 32 across all experiments.
- Training epochs: The model is trained for 1,000 epochs to ensure full convergence of the network parameters.

As shown in Table 1, to ensure reproducibility and fair comparison, we provide detailed information on the datasets, data splits, preprocessing pipeline, RAW normalization procedure, input resolution, and data augmentation strategy used in our experiments. In addition to the Zurich RAW-to-DSLR dataset used in the main comparison, we further introduced the MIT-Adobe FiveK and SR-RAW datasets for cross-dataset generalization evaluation:

- The MIT-Adobe FiveK dataset consists of RAW photographs acquired via DSLR cameras, serving as a prevalent benchmark for fundamental low-level vision applications, including image enhancement, exposure adjustment, and style transfer.
- Targeted at multiscale image restoration, the SR-RAW dataset provides authentic degraded RAW inputs to evaluate the efficacy of advanced RAW-domain reconstruction and super-resolution methodologies.
- Comprising mobile-sensor RAW images paired with high-fidelity DSLR reference counterparts, the Zurich RAW-to-DSLR dataset is extensively utilized for assessing RAW-to-RGB mapping pipelines, notably in learning-based ISP and visual enhancement.

**Table 1:** Dataset split and preprocessing protocol used for all compared methods.

Dataset	Total samples	Train/test split
Zurich RAW-to-DSLR	48,043 paired patches	46,839/1,204
MIT-Adobe FiveK	5,000 RAW images	4,800/200
SR-RAW	7,293 paired samples	7,000/293

### 4.2 Training protocol and loss functions

To ensure a fair and transparent comparison, the baseline models and the proposed EQISP were trained using different loss settings according to their respective original designs. Specifically, each baseline model was optimized using the loss function adopted in its original formulation or commonly used implementation for image-to-image reconstruction. The EQISP-specific loss was applied only to the proposed EQISP model and was not used to optimize the baseline models.

For the proposed EQISP, a two-phase training strategy was adopted. In the first phase, the floating-point QCNN backbone was trained to obtain a stable RAW-to-RGB reconstruction capability. In the second phase, dynamic fixed-point quantization was introduced, and the model was fine-tuned using the EQISP-specific hierarchical reconstruction loss. For Level 4 and Level 5, the model was optimized using the mean squared error loss:


$$\begin{array}{c} L_{\text{MSE}}=\frac{1}{N}\overset{N}{\underset{i=1}{\sum }}{\left({ŷ}_{i}-y_{i}\right)}^{2} \end{array}$$ (30)

For Level 2 and Level 3, the loss combines VGG-based perceptual loss and MSE loss with a ratio of 4:1:


$$\begin{array}{c} L_{2,3}=0.8L_{\text{VGG}}+0.2L_{\text{MSE}} \end{array}$$ (31)

For Level 1, the final reconstruction loss is defined as:


$$\begin{array}{c} L_{1}=L_{\text{VGG}}+0.75L_{\text{SSIM}}+0.05L_{\text{MSE}} \end{array}$$ (32)

where $L_{\text{SSIM}}=1-\text{SSIM}\left(Ŷ,Y\right)$ (Equations 30–32), Ŷ denotes the reconstructed RGB image, and *Y* denotes the DSLR reference image.

The training losses used for the compared methods are summarized in Table 2. Importantly, the EQISP-specific hierarchical loss was not applied to the baseline models, thereby avoiding potential advantages or disadvantages caused by changing their original optimization objectives.

**Table 2:** Comparisons of image quality metrics and computational complexity across two datasets.

Dataset	Method	PSNR↑	SSIM↑	GFLOPs↓
MIT-Adobe FiveK	Pix2Pix	19.02	0.8165	482.481
	U-Net	20.31	0.8462	482.501
	PyNET	21.15	0.8527	692.461
	EQISP (our)	**21.83**	**0.8901**	**156.294**
SR-RAW	Pix2Pix	19.94	0.8315	501.294
	U-Net	19.26	0.8395	472.384
	PyNET	20.07	0.8462	621.713
	EQISP (our)	**21.02**	**0.8716**	**162.527**

Bold values indicate the best experimental results.

### 4.3 Ablation study

**Figure 3:**
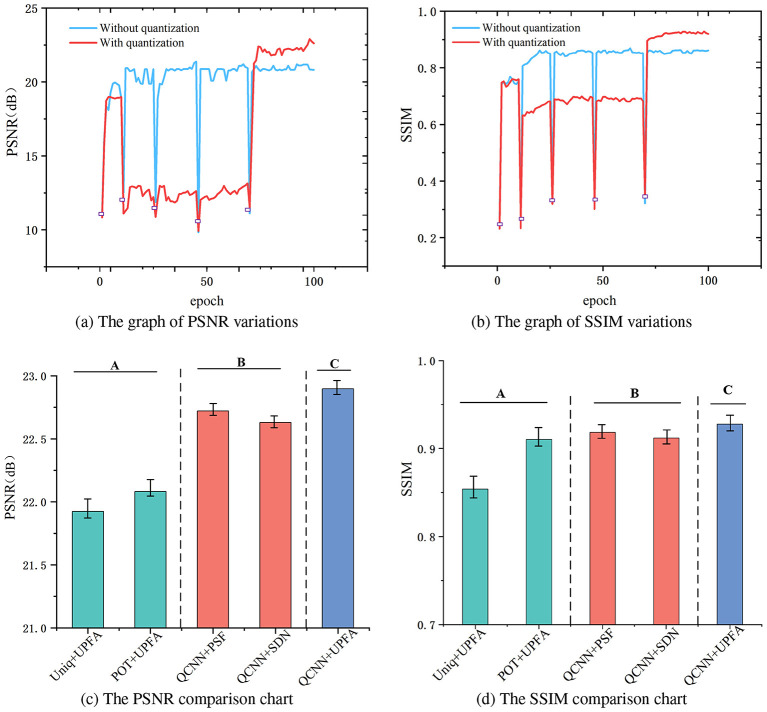
Training dynamics and ablation comparisons of QCNN and UPFA. **(a)** PSNR curves during training, where the blue curve denotes the model without quantization and the red curve denotes the model with quantization. **(b)** SSIM curves during training under the same two settings. **(c)** PSNR comparison of different component combinations. Group A compares alternative quantization strategies combined with UPFA, including Uniq+UPFA and POT+UPFA. Group B compares alternative fusion strategies combined with QCNN, including QCNN+PSF and QCNN+SDN. Group C denotes the complete proposed model, QCNN+UPFA. **(d)** SSIM comparison under the same grouping strategy as in **(c)**. **(a)** The graph of PSNR variations. **(b)** The graph of SSIM variations. **(c)** The PSNR comparison chart. **(d)** The SSIM comparison chart.

To evaluate the effectiveness of the UPFA and QCNN components, we conduct a comprehensive comparison with several state-of-the-art techniques on Zurich dataset. The detailed numerical metrics are summarized in Table 3, we compare uniform quantization (Baskin et al., 2021), POT quantization (Li et al., 2019), and QCNN to assess their performance in the proposed method. Uniform quantization uses the same bit-width across all network parameters. This approach can introduce inefficiencies or degrade accuracy, as layers vary in sensitivity to quantization errors. In contrast, POT quantization limits scaling factors to integer powers of two. This may not align well with parameter distributions, leading to suboptimal performance. QCNN uses advanced statistical techniques to determine optimal fixed-point formats for each network layer. This enables precise control of quantization errors and improves overall model performance. As shown in Figure 3c, QCNN consistently outperforms both POT and uniform quantization in key metrics, such as accuracy and computational efficiency. We also compare the PSF gradient domain fusion algorithm (Zhang and Ma, 2021) and the SDN weighted average fusion algorithm (Tang et al., 2023) with the proposed UPFA method. In high-contrast areas, the PSF algorithm often introduces unnatural artifacts due to gradient adjustments. It also requires complex post-processing to maintain gradient coherence, increasing computational complexity. Under extreme exposure conditions, the SDN algorithm often loses significant details due to underexposure or overexposure. This adversely affects image quality. In contrast, UPFA processes images at multiple scales, capturing both coarse and fine details simultaneously. It also preserves edge information during fusion, preventing blurring and enhancing output sharpness. As shown in Figure 3d, UPFA significantly outperforms both PSF and SDN in image quality, preserving details while maintaining sharpness and minimizing blurring.

**Table 3:** Quantitative ablation study with different components in Zurich dataset.

QCNN	Uniq	POT	UPFA	PSF	SDN	PSNR ↑	SSIM ↑	LPIPS ↓	GFLOPs ↓
✗	✓	✗	✓	✗	✗	21.8361	0.8571	0.3977	286.7134
✗	✓	✗	✗	✓	✗	18.1026	0.6827	0.5902	263.4086
✗	✓	✗	✗	✗	✓	17.6201	0.6040	0.6018	273.0372
✗	✗	✓	✓	✗	✗	22.1864	0.9176	0.3180	318.7459
✗	✗	✓	✗	✓	✗	20.8735	0.7629	0.3472	296.3015
✗	✗	✓	✗	✗	✓	20.0325	0.7153	0.3912	301.7522
✓	✗	✗	✓	✗	✗	**22.9057**	**0.9278**	**0.2146**	**164.8430**

The ↑ indicates that the higher this metric is, the better the performance. The ↓ indicates that the lower the metric, the better the performance. The ✗ indicates that component is removed. Bold values indicate the best experimental results.

To clarify the training dynamics, we present the fluctuations in Peak signal to noise ratio(PSNR) and Multi-Scale Structural Similarity Index(SSIM) for the EQISP model during training, as shown in Figures 3a, b. Retraining Levels 2–5, along with Level 1, causes variations in performance metrics during the transition to training a new level. The initial layer extracts basic features from the raw input data, but this process risks discarding critical high-level information needed for accuracy in subsequent layers. Conversely, even minor quantization errors in the final layer can disproportionately affect accuracy by directly influencing the model's output. To address these issues, quantization is excluded from both the first and last layers of the EQISP model. The results show a significant decline in both PSNR and SSIM scores when quantization is applied to the intermediate layers. This decline is mainly due to changes in the numerical range during quantization, leading to the loss of crucial information. The observed performance decrease results from quantization altering the numerical range of the model's parameters, which may lead to the loss of important details necessary for accurate predictions. As training progresses to the final layer, both PSNR and SSIM scores stabilize, suggesting that the model converges to optimal performance despite earlier disruptions caused by quantization.

### 4.4 Comparative experiment

To evaluate the effectiveness of the proposed method, we compare it with several state-of-the-art approaches in the field. Table 4 presents a detailed comparison of PSNR, SSIM, LPIPS, and the number of giga floating-point operations (GFLOPs) for EQISP and various benchmark methods. For the comparative experiments, all methods were evaluated under the same dataset split, input resolution, and evaluation metrics. The baseline models were trained using their original loss functions or standard reconstruction objectives, as summarized in Table 2. The EQISP-specific hierarchical loss was used only for the proposed EQISP model during the quantization-aware fine-tuning stage. Therefore, the baseline models were not optimized with EQISP-specific objectives, which ensures that the comparison does not introduce additional advantages or disadvantages by modifying the original training objectives of the competing methods. EQISP consistently outperforms competing models in both PSNR and SSIM, indicating superior image quality. This superior performance is achieved while maintaining lower computational costs, thanks to the quantization process implemented by EQISP.

**Table 4:** Comparisons of experimental results in ISP tasks among numerous deep learning methods on test data.

Method	LPIPS↓	PSNR↑	SSIM↑	GFLOPs↓
SPADE (Park et al., 2019)	0.2980	20.96	0.8586	592.557G
PyNET (Ignatov et al., 2020)	0.3371	21.19	0.8620	696.247G
U-Net (Ronneberger et al., 2015)	0.3014	20.81	0.8545	492.783G
Pix2Pix (Isola et al., 2017)	0.3732	20.93	0.8532	452.212G
SRGAN (Ledig et al., 2017)	0.3875	20.06	0.8501	508.745G
VDSR (Kim et al., 2016)	0.4261	19.78	0.8457	433.347G
SRCNN (Dong et al., 2015)	0.4852	18.56	0.8268	409.218G
EQISP	0.2146	22.90	0.9278	164.843G

The ↑ indicates that the higher this metric is, the better the performance. The ↓ indicates that the lower the metric, the better the performance. Bold values indicate the best experimental results.

**Figure 4:**
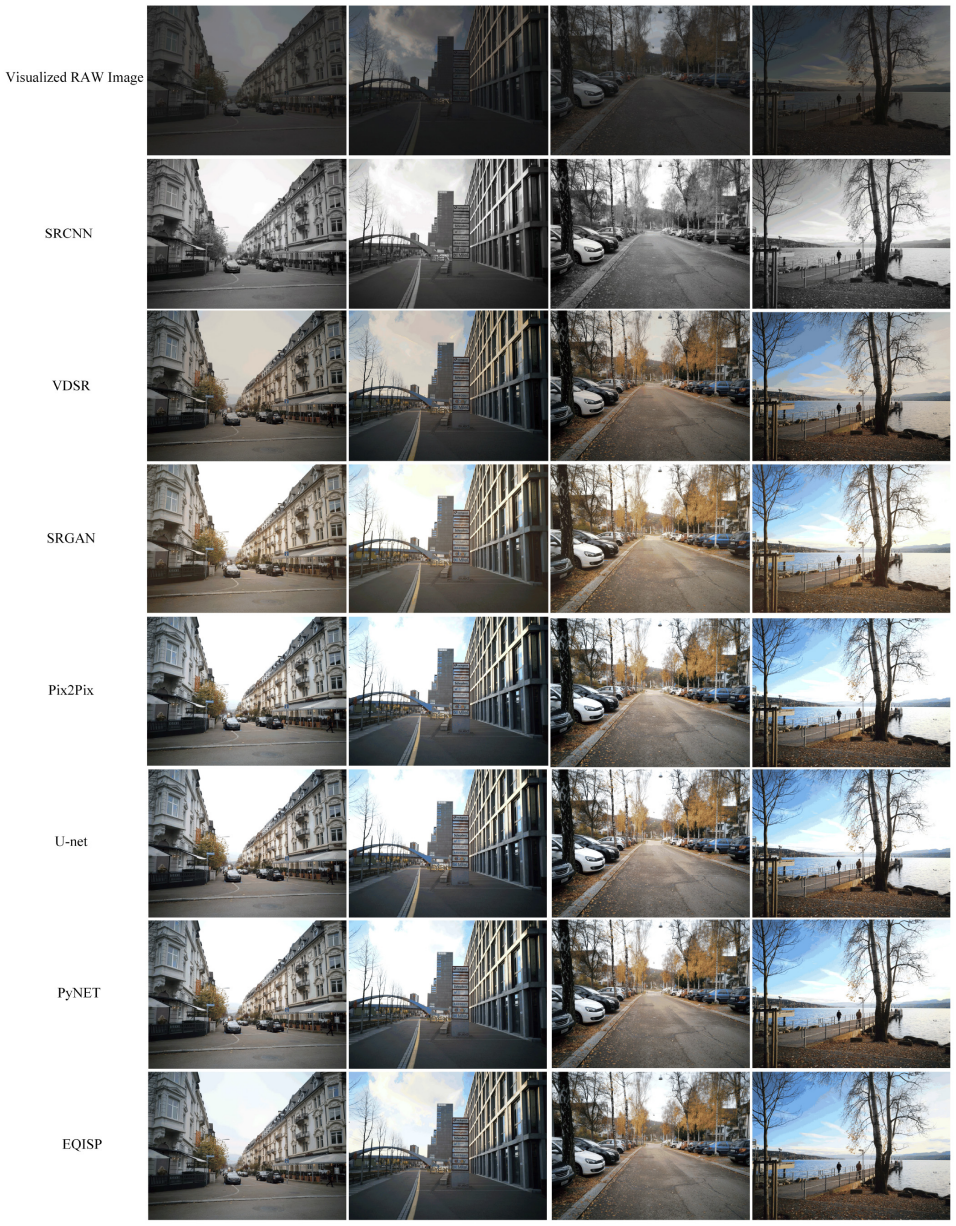
A comparative visualization of the visual effects between EQISP and other models.

**Figure 5:**
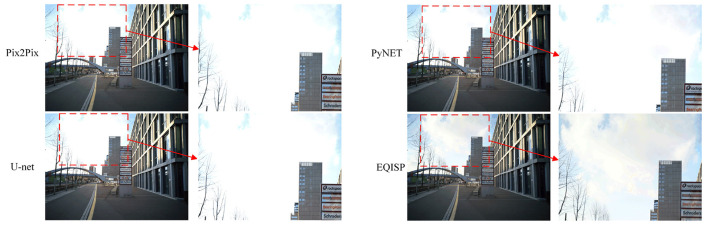
The local detail comparison images of EQISP with Pix2Pix, U-Net, and PyNET.

SPADE's complex architecture, featuring spatially adaptive modulation, imposes high computational demands and training instability across diverse datasets, compounded by its incompatibility with variable-resolution evaluations due to fixed input dimensions. PyNET requires extensive preprocessing and parameter tuning for generalization under varying imaging conditions, yielding the highest GFLOPs and highlighting inefficiency. Pix2Pix's L1 loss and conditional GANs prioritize low-frequency components and global structure, often resulting in blurred images, softened edges, mode collapse, and limited output diversity. U-Net's symmetric encoder-decoder design effectively captures local features but encounters downsampling-upsampling bottlenecks, leading to artifacts in raw-to-RGB transformations under varying illumination or noise, irreversible loss of spatial hierarchies, and inferior metrics. SRGAN's adversarial training enhances perceptual quality yet induces instability, artifacts, unnatural textures, hallucinations, and distortions in high-dynamic-range images, reflected in suboptimal PSNR and elevated overhead. VDSR's single-scale residual network inadequately handles demosaicing and denoising, amplifying noise, producing desaturated colors, and yielding poor chromatic restoration and metrics. SRCNN, an early shallow CNN, lacks representational depth, failing to capture intricate features and high-frequency details; its bicubic upscaling introduces blurring artifacts, compromising perceptual quality, color fidelity, and resulting in the lowest PSNR and SSIM scores. As evidenced in Table 4 and visual comparisons in Figure 4, EQISP consistently outperforms baselines in PSNR, SSIM, and GFLOPs through multi-scale integration and optimized quantization, balancing efficiency with reconstruction fidelity. As illustrated in Figure 5, magnified reconstructions from Pix2Pix, PyNET, and U-Net compared to EQISP highlight EQISP's superior ability to capture cloud formations and textures. In challenging atmospheric and illumination scenarios, EQISP more robustly preserves structural integrity and details, thereby underscoring its efficacy in remote sensing applications.

**Figure 6:**
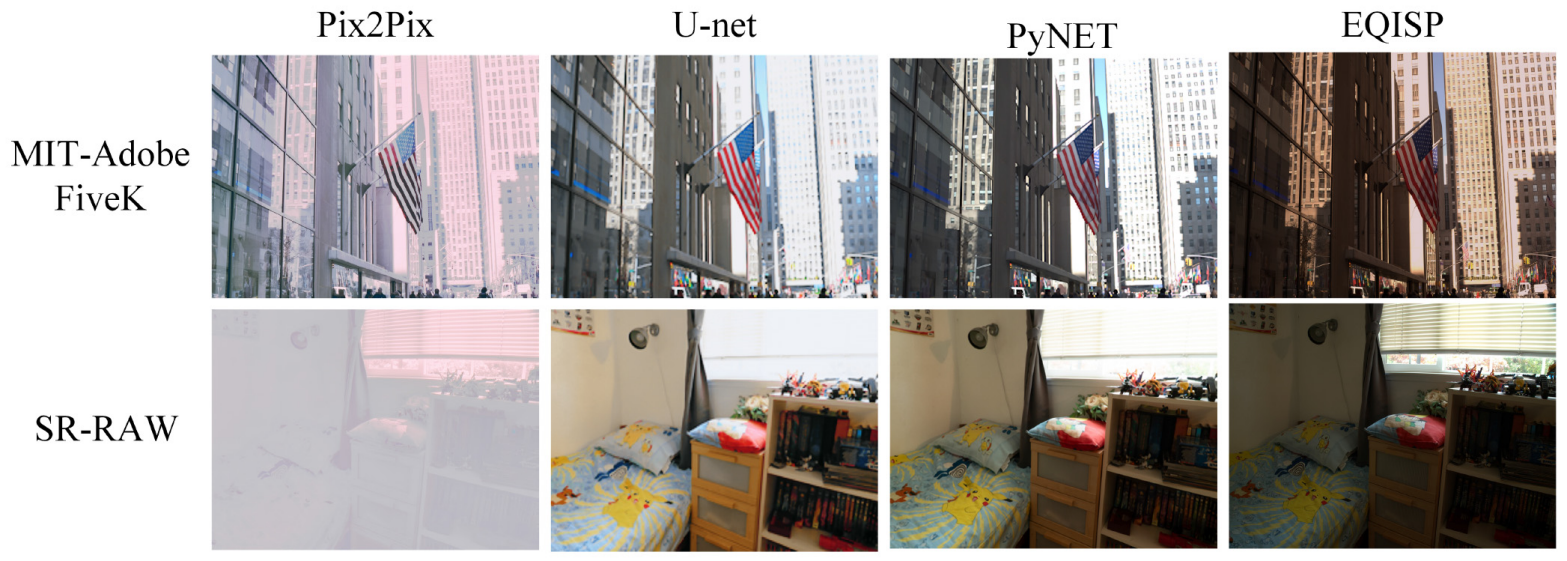
Qualitative comparison of Pix2Pix, U-Net, PyNET, and EQISP on the MIT-Adobe FiveK and SR-RAW datasets.

To further verify the generalizability of the proposed EQISP beyond the Zurich RAW-to-DSLR dataset, we conducted additional comparative experiments on two datasets, namely MIT-Adobe FiveK and SR-RAW. These datasets contain different scene contents and imaging conditions from the Zurich dataset, thereby providing a broader evaluation of whether the performance gains of EQISP remain consistent across diverse data distributions. For a fair comparison, all competing methods were evaluated using the same dataset split, pre-processing pipeline, input resolution setting, and evaluation metrics within each dataset. Table 2 reports the quantitative comparison results on the MIT-Adobe FiveK and SR-RAW datasets. On the MIT-Adobe FiveK dataset, EQISP achieves the best PSNR and SSIM values, reaching 21.83 dB and 0.8901, respectively, while requiring only 156.294 GFLOPs. Compared with PyNET, which obtains the strongest baseline PSNR of 21.15 dB, EQISP improves PSNR by 0.68 dB and reduces computational complexity from 692.410 GFLOPs to 156.294 GFLOPs. On the SR-RAW dataset, EQISP also achieves the best PSNR and SSIM values, reaching 21.02 dB and 0.8716, respectively, with 162.527 GFLOPs. Compared with PyNET, EQISP improves PSNR by 0.95 dB and reduces computational complexity by approximately 3.83 times. The qualitative results in Figure 6 further show that EQISP produces more balanced visual reconstruction across different scenes. In the MIT-Adobe FiveK example, EQISP better preserves structural contrast and avoids the washed-out appearance observed in some baseline outputs. In the SR-RAW example, EQISP maintains a better balance between highlight regions and shadow details, whereas some baseline methods suffer from overexposure, low contrast, or insufficient detail restoration. These results indicate that the proposed quantization and pyramid fusion strategy is not limited to the Zurich RAW-to-DSLR dataset, but maintains consistent advantages across additional datasets with different visual characteristics.

### 4.5 Edge deployment evaluation

To further substantiate the deployment efficiency of EQISP on realistic edge hardware, we conducted an additional inference experiment on an NVIDIA Jetson TX2 development board using the Zurich RAW-to-DSLR dataset. Unlike the training experiments performed on an NVIDIA Tesla V100 GPU, this evaluation focuses on deployment-oriented metrics that are more directly related to resource-constrained embodied perception, including model size, inference latency, inference frames per second (FPS), and peak memory usage.

As shown in Table 5, EQISP achieves the smallest model size among the compared methods, requiring only 57 MB, compared with 128 MB for PyNET and 94 MB for Pix2Pix. In terms of runtime efficiency, EQISP achieves an inference latency of 189 ms and an inference speed of 6.1 FPS on the Jetson TX2 platform, substantially outperforming PyNET and Pix2Pix, whose latencies are 446 ms and 397 ms, respectively. Moreover, EQISP requires only 2.2 GB of peak memory, which is lower than the 4.0 GB required by PyNET and the 3.8 GB required by Pix2Pix. These results demonstrate that the proposed quantization-based design not only reduces theoretical computational complexity, as reflected by GFLOPs, but also translates into practical improvements in latency, memory footprint, and inference throughput on an actual edge device. Therefore, the additional deployment experiment provides direct evidence supporting the suitability of EQISP for resource-constrained embodied perception scenarios.

**Table 5:** Performance comparison on the NVIDIA Jetson TX2 development board using the Zurich RAW-to-DSLR dataset.

Model	Model size(MB)	Latency (ms)	Inference FPS	Peak memory (GB)
PyNET	128	446	3.0	4.0
Pix2Pix	94	397	2.9	3.8
EQISP	**57**	**189**	**6.1**	**2.2**

Bold values indicate the best experimental results.

## 5 Conclusion

Efficient image signal processing was essential for resource-constrained visual perception in autonomous robots and embodied intelligent systems. To address the computational complexity and detail loss associated with CNN-based ISP models, this study proposed EQISP, an enhanced quantized image signal processor for low-complexity and high-quality RAW-to-RGB reconstruction. EQISP integrated QCNN and UPFA to balance computational efficiency and reconstruction fidelity. QCNN employed dynamic fixed-point hybrid quantization to reduce computational load by adaptively adjusting the numerical range of network parameters, whereas UPFA combined Gaussian and Laplacian pyramids to preserve global illumination and fine details. Experimental results showed that EQISP reduced computational load by 1.21–4.24 times compared with existing methods and achieved a PSNR of 22.90 dB and an SSIM of 0.9278. Compared with state-of-the-art ISP methods, EQISP demonstrated superior quantitative performance and visual reconstruction quality, particularly in preserving image details and overall structure. In addition, deployment evaluation on an NVIDIA Jetson TX2 development board further verified the practical efficiency of EQISP, with a model size of 57 MB, an inference latency of 189 ms, an inference speed of 6.1 FPS, and a peak memory usage of 2.2 GB. These results demonstrate that EQISP is not only computationally efficient in terms of GFLOPs but also practically deployable on edge platforms with constrained memory and computational resources, making it a promising visual front end for autonomous robots and embodied intelligent systems.

## Data Availability

The raw data supporting the conclusions of this article will be made available by the authors, without undue reservation.

## References

[B1] Allwin P. S. Gomony M. D. Geilen M. (2024). “Run-time non-uniform quantization for dynamic neural networks in wireless communication,” in 2024 29th Asia and South Pacific Design Automation Conference (ASP-DAC) (IEEE), 915–920. doi: 10.1109/ASP-DAC58780.2024.10473894

[B2] Ashkboos S. Verhoef B. Hoefler T. Eleftheriou E. Dazzi M. (2024). Efqat: an efficient framework for quantization-aware training. arXiv preprint arXiv:2411.11038.

[B3] Azuma S.-I. Sugie T. (2008a). Optimal dynamic quantizers for discrete-valued input control. Automatica 44, 396–406. doi: 10.1016/j.automatica.2007.06.012

[B4] Azuma S.-I. Sugie T. (2008b). Synthesis of optimal dynamic quantizers for discrete-valued input control. IEEE Trans. Automat. Contr. 53, 2064–2075. doi: 10.1109/TAC.2008.929400

[B5] Baskin C. Liss N. Schwartz E. Zheltonozhskii E. Giryes R. Bronstein A. M. . (2021). Uniq: uniform noise injection for non-uniform quantization of neural networks. ACM Trans. Comput. Syst. 37, 1–15. doi: 10.1145/3444943

[B6] Bengio Y. (2012). “Deep learning of representations for unsupervised and transfer learning,” in Proceedings of ICML Workshop on Unsupervised and Transfer Learning (JMLR Workshop and Conference Proceedings), 17–36.

[B7] Biswas A. KS G,. R. Patel M. S. Prasad B. P. (2020). “Fast multi-stage motion-compensated approach for hdr,” in 2020 IEEE International Conference on Image Processing (ICIP) (IEEE), 2980–2984. doi: 10.1109/ICIP40778.2020.9190926

[B8] Bondarenko Y. Del Chiaro R. Nagel M. (2024). Low-rank quantization-aware training for llms. arXiv preprint arXiv:2406.06385.

[B9] Catley-Chandar S. Tanay T. Vandroux L. Leonardis A. Slabaugh G. Pérez-Pellitero E. (2022). Flexhdr: modeling alignment and exposure uncertainties for flexible hdr imaging. IEEE Trans. Image Proc. 31, 5923–5935. doi: 10.1109/TIP.2022.3203562 36074867

[B10] Chen H. (2022). Knowledge distillation with error-correcting transfer learning for wind power prediction. arXiv preprint arXiv:2204.00649.

[B11] Chen R. Zheng B. Zhang H. Chen Q. Yan C. Slabaugh G. . (2023). “Improving dynamic hdr imaging with fusion transformer,” in Proceedings of the AAAI Conference on Artificial Intelligence, 340–349. doi: 10.1609/aaai.v37i1.25107

[B12] Chen X. Liu Y. Zhang Z. Qiao Y. Dong C. (2021). “Hdrunet: single image hdr reconstruction with denoising and dequantization,” in Proceedings of the IEEE/CVF Conference on Computer Vision and Pattern Recognition, 354–363. doi: 10.1109/CVPRW53098.2021.00045

[B13] Dong C. Loy C. C. He K. Tang X. (2015). Image super-resolution using deep convolutional networks. IEEE Trans. Pattern Anal. Mach. Intell. 38, 295–307. doi: 10.1109/TPAMI.2015.2439281 26761735

[B14] Fan W. Yang Y. Qi J. Zhang Q. Liao C. Wen L. . (2024). A deep-learning-based framework for identifying and localizing multiple abnormalities and assessing cardiomegaly in chest x-ray. Nat. Commun. 15:1347. doi: 10.1038/s41467-024-45599-z 38355644 PMC10867134

[B15] Farooq J. Bazaz M. A. (2020). A novel adaptive deep learning model of COVID-19 with focus on mortality reduction strategies. Chaos, Solit. Fractals 138:110148. doi: 10.1016/j.chaos.2020.110148 PMC7373073 32834586

[B16] Gou J. Yu B. Maybank S. J. Tao D. (2021). Knowledge distillation: a survey. Int. J. Comput. Vis. 129, 1789–1819. doi: 10.1007/s11263-021-01453-z

[B17] Gupta A. Anpalagan A. Guan L. Khwaja A. S. (2021). Deep learning for object detection and scene perception in self-driving cars: survey, challenges, and open issues. Array 10:100057. doi: 10.1016/j.array.2021.100057

[B18] Hu X. Shen L. Jiang M. Ma R. An P. (2022). La-hdr: light adaptive HDR reconstruction framework for single ldr image considering varied light conditions. IEEE Trans. Multim. 25, 4814–4829. doi: 10.1109/TMM.2022.3183404

[B19] Ignatov A. Van Gool L. Timofte R. (2020). “Replacing mobile camera isp with a single deep learning model,” in Proceedings of the IEEE/CVF Conference on Computer Vision and Pattern Recognition Workshops, 536–537. doi: 10.1109/CVPRW50498.2020.00276

[B20] Isola P. Zhu J.-Y. Zhou T. Efros A. A. (2017). “Image-to-image translation with conditional adversarial networks,” in Proceedings of the IEEE Conference on Computer Vision and Pattern Recognition, 1125–1134. doi: 10.1109/CVPR.2017.632

[B21] Jacob B. Kligys S. Chen B. Zhu M. Tang M. Howard A. . (2018). “Quantization and training of neural networks for efficient integer-arithmetic-only inference,” in Proceedings of the IEEE Conference on Computer Vision and Pattern Recognition, 2704–2713. doi: 10.1109/CVPR.2018.00286

[B22] Kang J. Gwak J. (2020). Ensemble learning of lightweight deep learning models using knowledge distillation for image classification. Mathematics 8:1652. doi: 10.3390/math8101652

[B23] Keerativittayanun S. Kondo T. Kotani K. Phatrapornnant T. Karnjana J. (2021). Two-layer pyramid-based blending method for exposure fusion. Mach. Vis. Appl. 32:48. doi: 10.1007/s00138-021-01175-9

[B24] Kim J. Lee J. K. Lee K. M. (2016). “Accurate image super-resolution using very deep convolutional networks,” in Proceedings of the IEEE Conference on Computer Vision and Pattern Recognition, 1646–1654. doi: 10.1109/CVPR.2016.182

[B25] Kong L. Li B. Xiong Y. Zhang H. Gu H. Chen J. (2024). “Safnet: selective alignment fusion network for efficient hdr imaging,” in European Conference on Computer Vision (Springer), 256–273. doi: 10.1007/978-3-031-73347-5_15

[B26] Ledig C. Theis L. Huszár F. Caballero J. Cunningham A. Acosta A. . (2017). “Photo-realistic single image super-resolution using a generative adversarial network,” in Proceedings of the IEEE Conference on Computer Vision and Pattern Recognition, 4681–4690. doi: 10.1109/CVPR.2017.19

[B27] Li F. Hou W. Jia P. (2024). Rmfa-net: a neural ISP for real raw to rgb image reconstruction. arXiv preprint arXiv:2406.11469.

[B28] Li Y. Dong X. Wang W. (2019). Additive powers-of-two quantization: an efficient non-uniform discretization for neural networks. arXiv preprint arXiv:1909.13144.

[B29] Liaw Y.-C. Lo W. Lai J. Z. (2002). Image restoration of compressed image using classified vector quantization. Pattern Recognit. 35, 329–340. doi: 10.1016/S0031-3203(01)00048-6

[B30] Liu S. Zhang X. Sun L. Liang Z. Zeng H. Zhang L. (2023a). “Joint hdr denoising and fusion: A real-world mobile hdr image dataset,” in Proceedings of the IEEE/CVF Conference on Computer Vision and Pattern Recognition, 13966–13975. doi: 10.1109/CVPR52729.2023.01342

[B31] Liu Z. Oguz B. Zhao C. Chang E. Stock P. Mehdad Y. . (2023b). Llm-qat: data-free quantization aware training for large language models. arXiv preprint arXiv:2305.17888.

[B32] Oh S. Sim H. Kim J. Lee J. (2022). “Non-uniform step size quantization for accurate post-training quantization,” in European Conference on Computer Vision (Springer), 658–673. doi: 10.1007/978-3-031-20083-0_39

[B33] Oh T.-H. Lee J.-Y. Tai Y.-W. Kweon I. S. (2014). Robust high dynamic range imaging by rank minimization. IEEE Trans. Pattern Anal. Mach. Intell. 37, 1219–1232. doi: 10.1109/TPAMI.2014.2361338 26357344

[B34] Park T. Liu M.-Y. Wang T.-C. Zhu J.-Y. (2019). “Semantic image synthesis with spatially-adaptive normalization,” in Proceedings of the IEEE/CVF Conference on Computer Vision and Pattern Recognition, 2337–2346. doi: 10.1109/CVPR.2019.00244

[B35] Patel S. Androutsos D. Kyan M. (2015). “Adaptive exposure fusion for high dynamic range imaging,” in 2015 IEEE International Conference on Image Processing (ICIP) (IEEE), 4679–4683. doi: 10.1109/ICIP.2015.7351694

[B36] Ren D. Li W. Ding T. Wang L. Fan Q. Huo J. . (2025). Onnxpruner: Onnx-based general model pruning adapter. IEEE Trans. Pattern Anal. Mach. Intell. 47, 5806–5817. doi: 10.1109/TPAMI.2025.3554560 40131753

[B37] Ronneberger O. Fischer P. Brox T. (2015). “U-net: convolutional networks for biomedical image segmentation,” in Medical image computing and computer-assisted intervention-MICCAI 2015: 18th international conference, Munich, Germany, October 5–9, 2015, proceedings, part III 18 (Springer), 234–241. doi: 10.1007/978-3-319-24574-4_28

[B38] Santos C. F. G. D. Arrais R. R. Silva J. V. S. D. Silva M. H. M. D. Neto W. B. G. D. A. Lopes L. T. . (2025). Isp meets deep learning: a survey on deep learning methods for image signal processing. ACM Comput. Surv. 57, 1–44. doi: 10.1145/3708516

[B39] Shen M. Liang F. Gong R. Li Y. Li C. Lin C. . (2021). “Once quantization-aware training: High performance extremely low-bit architecture search,” in Proceedings of the IEEE/CVF International Conference on Computer Vision, 5340–5349. doi: 10.1109/ICCV48922.2021.00529

[B40] Sze V. Chen Y.-H. Yang T.-J. Emer J. S. (2017). Efficient processing of deep neural networks: a tutorial and survey. Proc. IEEE 105, 2295–2329. doi: 10.1109/JPROC.2017.2761740

[B41] Tan C. Sun F. Kong T. Zhang W. Yang C. Liu C. (2018). “A survey on deep transfer learning,” in Artificial Neural Networks and Machine Learning-ICANN 2018: 27th International Conference on Artificial Neural Networks, Rhodes, Greece, October 4–7, 2018, Proceedings, Part III 27 (Springer), 270–279. doi: 10.1007/978-3-030-01424-7_27

[B42] Tang L. Zhang H. Xu H. Ma J. (2023). Rethinking the necessity of image fusion in high-level vision tasks: a practical infrared and visible image fusion network based on progressive semantic injection and scene fidelity. Inf. Fusion 99:101870. doi: 10.1016/j.inffus.2023.101870

[B43] Wang Y. Xu T. Zhang F. Xue T. Gu J. (2024). “Adaptiveisp: learning an adaptive image signal processor for object detection,” in Advances in Neural Information Processing Systems.

[B44] Wu C.-T. Isikdogan L. F. Rao S. Nayak B. Gerasimow T. Sutic A. . (2019). “Visionisp: repurposing the image signal processor for computer vision applications,” in 2019 IEEE International Conference on Image Processing (ICIP) (IEEE), 4624–4628. doi: 10.1109/ICIP.2019.8803607

[B45] Wu J. Leng C. Wang Y. Hu Q. Cheng J. (2016). “Quantized convolutional neural networks for mobile devices,” in Proceedings of the IEEE Conference on Computer Vision and Pattern Recognition, 4820–4828. doi: 10.1109/CVPR.2016.521

[B46] Wu N. Wang H. (2018). Deep learning adaptive dynamic programming for real time energy management and control strategy of micro-grid. J. Clean. Prod. 204, 1169–1177. doi: 10.1016/j.jclepro.2018.09.052

[B47] Yang Y. Wu J. Huang S. Lin P. (2019). Multiexposure estimation and fusion based on a sparsity exposure dictionary. IEEE Trans. Instrum. Meas. 69, 4753–4767. doi: 10.1109/TIM.2019.2951864

[B48] Zhang H. Ma J. (2021). Sdnet: a versatile squeeze-and-decomposition network for real-time image fusion. Int. J. Comput. Vis. 129, 2761–2785. doi: 10.1007/s11263-021-01501-8

